# Studying ferroptosis and iron metabolism pre- and post-radiotherapy treatment in breast cancer patients

**DOI:** 10.1186/s43046-023-00162-7

**Published:** 2023-02-27

**Authors:** Sanaa A. El-Benhawy, Ibrahim G. Abdelrhman, Nadia A. Sadek, Enayat I. Fahmy, Ahmed A. AboGabal, Hossam Elmasry, Sally A. M. Saleh, Ola A. Sakr, Mona Nagy Elwany, Maha Abubakr Feissal Rabie

**Affiliations:** 1 Radiation Sciences Department, Medical Research Institute, Alexandria University, Alexandria, Egypt; 2 Radiology and Medical Imaging Department, Faculty of Applied Health Sciences, October 6 University, Cairo, Egypt; 3 Hematology Department, Medical Research Institute, Alexandria University, Alexandria, Egypt; 4 Radiation Oncology Department, National Cancer Institute, Cairo University, Cairo, Egypt; 5 Medical Laboratory Specialist, Baheya Foundation for Early Detection and Treatment of Breast Cancer, Cairo, Egypt; 6 Cancer Management and Research Department, Medical Research Institute, Alexandria University, Alexandria, Egypt; 7 Pathology Department, Medical Research Institute, Alexandria University, Alexandria, Egypt; 8 Medical Laboratory Department, Faculty of Applied Health Science Technology, Pharos University, Alexandria, Egypt

**Keywords:** Radiotherapy, Ferroptosis, Breast cancer, Iron profile

## Abstract

**Background:**

Radiotherapy (RT) is an important part of the treatment of many tumors. Radiotherapy causes oxidative damage in all cellular compartments, including lipid membrane, on a random basis. Toxic lipid peroxidation accumulation has only lately been linked to a regulated type of cell death known as ferroptosis. Iron is required for ferroptosis sensitization in cells.

**Aim of the work:**

This work aimed to study ferroptosis and iron metabolism before and after RT in BC patients.

**Subjects and methods:**

Eighty participants were included divided into two main groups: group I: 40 BC patients treated with RT. Group II: 40 healthy volunteers’ age and sex matched as control group. Venous blood samples were collected from BC patients (prior to and after RT) and healthy controls. Glutathione (GSH), malondialdehyde (MDA), serum iron levels and % of transferrin saturation were measured by colorimetric technique. Ferritin, ferroportin, and prostaglandin-endoperoxide synthase 2 (PTGS2) levels were assessed by ELISA.

**Results:**

Serum ferroportin, reduced glutathione, and ferritin showed significant decrease after radiotherapy in comparison to before radiotherapy. However, there was significant increase in serum PTGS2, MDA, % of transferrin saturation and iron levels after radiotherapy in comparison to before radiotherapy.

**Conclusion:**

Radiotherapy induced ferroptosis in breast cancer patients as a new cell death mechanism and PTGS2 is a biomarker of ferroptosis. Iron modulation is a useful approach for the treatment of BC especially if combined with targeted therapy and immune-based therapy. Further studies are warranted to be translated into clinical compounds.

## Introduction

Radiotherapy (RT) is an important part of the treatment of many tumors. RT stochastically causes oxidative damage in all cellular compartments, including the lipid membrane. Toxic lipid peroxidation accumulation has only lately been linked to the direct cause of ferroptosis, a controlled form of cell death [1, 2].

Ferroptosis is an iron-dependent form of controlled cell death with properties distinct from other forms of cell death. The confluence of lipid, amino acid and iron metabolism is necessary for ferroptosis activation [3, 4]. The presence of redox-active iron, oxidation of polyunsaturated fatty acid (PUFA) containing phospholipids, and deficient or blocked lipid peroxide repair pathways are all hallmarks of ferroptosis [5].

The process of lipid peroxidation is triggered by free radicals that primarily affects the cell membrane unsaturated fatty acids. Initial reactive aldehydes (e.g., malondialdehyde (MDA)) and lipid hydroperoxides (LOOHs) are lipid peroxidation products that rise during ferroptosis [6, 7] as well as prostaglandin-endoperoxide synthase 2 (PTGS2) [2]. It was reported that PTGS2 is a pharmacodynamic biomarker of ferroptosis [8].

In mammals, reduced glutathione (GSH) is the main intracellular antioxidant [1]. Its depletion and the inactivation of glutathione peroxidase 4 (GPX4) are essential for initiation of ferroptosis [9].

The accumulation of excessive intracellular iron is essential for cell ferroptosis sensitization. Iron transport mechanisms normally maintain a proper balance of intracellular iron. The circulating glycoprotein transferrin (TF) can transport extracellular iron. The iron–protein complex (mainly ferritin) is used to store and transport imported iron. Ferroportin (FPN), the only known iron exporter that regulates iron efflux in mammals, may export intracellular iron [10, 11].

The majority of cancer cells have an abnormal iron metabolism and a high intracellular iron concentration. Cell development and proliferation are aided by iron. On the other hand, it could be involved in the Fenton reaction, which produces reactive oxygen species (ROS). Intracellular ROS may cause lipid peroxidation, which is required for ferroptosis to occur [9]. Iron appears to be a trigger for ferroptosis or a component of a crucial regulator. Thus, iron chelators can prevent the occurrence of ferroptosis. The metabolism of iron is divided into three stages: absorption, storage, and efflux. As a result, genes involved in iron metabolism may modulate intracellular iron concentration and mediate the process of ferroptosis [12]. In cancer cells, ionizing radiation causes ferroptosis. Ionizing radiation could cause ferroptosis in cancer cells since both IR and ferroptosis are linked to ROS. In cancer cells, IR increased total ROS. The buildup of lipid peroxidation is a characteristic of ferroptosis [2]. Therefore, the current study was undertaken to explore ferroptosis and iron metabolism before and after RT in breast cancer (BC) patients.

### Subjects and methods

This study included 80 subjects divided into two groups:

- Group I: 40 BC patients treated with RT.
- Group II: 40 healthy females as a control group, matched for age and menopausal status with the previous group.

Patients were selected from those admitted to Bahia Hospital, Cairo, Egypt. After diagnosis of malignancy, patients underwent surgery (modified radical mastectomy or conservative surgery) followed by pathological evaluation of the tumor included tumor type, grade, tumor size, numbers of axillary lymph nodes involved, and presence or absence of vascular invasion. Assessments of estrogen, progesterone receptors (ER, PR) and Her2/neu expression were also confirmed. After surgery, patients received chemotherapy protocol consists of 4 cycles of doxorubicin/cyclophosphamide (AC) followed by 4 cycles of paclitaxel. Radiotherapy protocol included daily irradiation dose of 2.67 Gy provided 5 days a week for 3 weeks, yielding a total dosage of 40 Gy/15 fractions. Written informed consent was obtained from all study subjects. Also, approval of the Research Ethics Committee of the Medical Research Institute (Ethics code: IORG0008812), Alexandria University, Egypt, was obtained prior to the study. All procedures performed in our study were in accordance with the ethical standards of our institution and national and with the 1975 Helsinki Declaration as revised in 2008.

### Inclusion and exclusion criteria

#### Patients group

Primary Females breast cancer patients. No previous history of any other type of cancer or chronic disorders. No history of blood transfusion. Metastatic patients, patients received hematinic drugs, and patients received radiotherapy before surgery are excluded from this study.

#### Control group

Healthy Females age matched with patients group with normal mammography findings and no previous history of cancer. No history of receiving any radiation therapy. No history of anemia or blood transfusion. Subjects received hematinic drugs and smokers are excluded from this study.

### Blood sample collection

Two venous blood samples (5 ml each) were collected from BC patients, one before and the second after completing RT. One venous blood sample (5 ml) was withdrawn from the normal healthy control subjects.

Blood sample was divided into two aliquots; one was added into EDTA containing tubes for determination of blood glutathione levels by colorimetric approach (Biodiagnostic, Egypt). The second aliquot was added in serum separating tubes. The blood sample was allowed to clot for 10–20 min at room temperature and centrifuged at 2000–3000 RPM for 20 min. The supernatants were carefully collected. Serum was stored at − 80 °C until used. Malondialdehyde (MDA) and serum iron levels were measured using commercially available local kits (Biodiagnostic, Egypt). Serum ferroportin and PTGS2 levels were assessed by ELISA technique according to manufacture protocol (Bioassay, China). Imbian-Ferritin ELISA kit (Imbian Lab, Russia) was used to measure serum ferritin.

Photometric color test was used for the quantitative determination of unsaturated iron binding capacity (UIBC) in human serum on Beckman Coulter analyzer.

$$\begin{array}{c}\mathrm{Total}\;\mathrm{Iron}\;\mathrm{Binding}\;\mathrm{Capacity}\;(\mathrm{TIBC})\;(\mathrm\mu\mathrm g/\mathrm{dl})\\\mathrm{TIBC}=\;\mathrm{Iron}\;+\;\mathrm{UIBC}\\\mathrm T\mathrm r\mathrm a\mathrm n\mathrm s\mathrm f\mathrm e\mathrm r\mathrm r\mathrm i\mathrm n\;\mathrm S\mathrm a\mathrm t\mathrm u\mathrm r\mathrm a\mathrm t\mathrm i\mathrm o\mathrm n\;\%=\;(\mathrm{Serum}\;\mathrm{iron}/\mathrm{TIBC})\;\times\;100\end{array}$$

### Statistical analyses

Data were fed to the computer and analyzed using IBM SPSS software package version 20.0. (Armonk, NY: IBM Corp.). The Kolmogorov–Smirnov test was used to verify the normality of distribution. Range (minimum and maximum), mean, and standard deviation were used to characterize quantitative data. Student’s *t* test was used for normally distributed quantitative variables to compare between two studied groups. Paired *t* test was used for normally distributed quantitative variables to compare between two periods. Mann–Whitney test was used for abnormally distributed quantitative variables to compare between two studied groups. Wilcoxon signed-ranks test was used for abnormally distributed quantitative variables to compare between two periods. Significance of the acquired results was assessed at a 5% level.

## Results

### Clinicopathological characteristics of BC patients

Clinicopathological characteristics of BC patients are illustrated in Table 1.

**Table 1:** Clinico-pathological characteristics of BC patients

	BC patients group (*n* = 40)
Age (years)	
Mean ± SD	42.52 ± 11.58 years
Range	(31–72) years
Histological grade	
I	3 (7.5%)
II	31 (77.5%)
III	6 (15%)
Axillary lymph node involvement	
Positive	19 (47.5%)
Negative	21 (52.5%)
ER status^a^	
Positive	34 (95%)
Negative	6 (5%)
PR status^b^	
Positive	37 (92.5%)
Negative	3 (7.5%)
Her-2/neu expression^c^	
Positive	0 (0%)
Negative	40 (100%)
Tumor type	
IDC^d^	35 (87.5%)
Other (ILC^e^ and MC^f^ and NST^g^)	5 (12.5%)
Type of surgery	
Breast conservation	13 (32.5%)
Mastectomy	27 (67.5%)

^a^Estrogen receptor status

^b^Progesterone receptor status

^c^Human epidermal growth factor receptor 2

^d^Invasive ductal carcinoma

^e^Invasive lobular carcinoma

^f^Mucinous carcinoma

^g^Invasive carcinoma of no special type

### Ferroptosis markers

#### PTGS2

Serum PTGS2 significantly increased in BCPs group either prior to or following RT in comparison to healthy volunteers (p1 = 0.002 and < 0.001, respectively). Moreover, this biomarker significantly increased after RT (p2 = 0.031) (Table 2).

**Table 2:** Statistical analysis of studied biomarkers in breast cancer patients

	Control group (*n* = 40)	Breast cancer patients (*n* = 40)	
		Before radiotherapy	After radiotherapy
PTGS2 (ng/ml)			
Range	1.07–2.89	1.53–40.0	1.38–45.0
Mean ± SD	2.27 ± 0.42	4.52 ± 2.06	5.68 ± 2.32
P1		0.002*	< 0.001*
P2		0.031*	
MDA (nmol/ml)			
Range	0.26–2.50	5.87–14.65	1.0–28.27
Mean ± SD	1.19 ± 0.66	6.65 ± 4.14	9.29 ± 6.26
P1		< 0.001*	< 0.001*
P2		0.026*	
GSH (mmole/L)			
Range	7.14–10.80	6.30–10.60	5.60–9.41
Mean ± SD	8.98 ± 0.88	8.49 ± 0.95	7.85 ± 0.96
P1		0.293	< 0.001*
P2		< 0.001*	
Ferroportin (ng/ml)			
Range	2.01–4.75	2.05–7.15	2.01–3.98
Mean ± SD	2.56 ± 0.59	3.48 ± 1.55	2.58 ± 0.99
P1		0.014*	0.740
P2		< 0.001^*^	
Hb (g/dl)			
Range	12.5 – 14.5	9.50–13.8	9.15–12.40
Mean ± SD	13.10 ± 2.81	11.40 ± 1.27	10.60 ± 0.89
P1		0.002*	< 0.001^*^
P2		0.149	
Iron (µg/dl)			
Range	29.0–106.0	22.0–113.0	33.0–111.0
Mean ± SD	61.73 ± 19.84	55.33 ± 19.45	66.64 ± 19.90
P1		0.177	0.305
P2		< 0.001*	
Ferritin (ng/ml)			
Range	8.30–86.3	6.10 – 140	8.70–97.9
Mean ± SD	40.99 ± 15.10	80.82 ± 22.0	59.91 ± 16.49
P1		0.028*	0.332
P2		< 0.001*	
Transferrin saturation (%)			
Range	5.74–37.60	5.54–32.03	8.27–33.50
Mean ± SD	18.54 ± 5.59	16.75 ± 5.85	19.59 ± 6.44
P1		0.197	0.476
P2		< 0.001*	
UIBC (µg/dl)			
Range	161.0–476.0	174.0–397.0	200.0–380.0
Mean ± SD	264.5 ± 60.88	281.0 ± 53.45	278.1 ± 48.43
P1		0.226	0.293
P2		0.582	
TIBC (µg/dl)			
Range	240.0–515.0	233.0–469.0	229.0–448.0
Mean ± SD	319.1 ± 68.84	336.4 ± 52.25	328.2 ± 48.23
P1		0.231	0.514
P2		0.099	

*P1 p* value for comparing between control group with BCPs either before or after RT

*P2 p* value for comparing between before and after radiotherapy

^*^Statistically significant at *p* ≤ 0.05

#### MDA

Serum MDA was significantly increased in BCPs group either prior to or following RT in comparison to healthy volunteers (p1 < 0.001 and < 0.001, respectively). Furthermore, MDA levels significantly increased after RT (p2 = 0.026) (Table 2).

#### Reduced GSH

Reduced GSH showed insignificant difference prior to RT in BCPs group and healthy volunteers (p1 = 0.293). However, this parameter significantly decreased after RT when compared to either before treatment or normal controls (p2 < 0.001 and p1 < 0.001, respectively) (Table 2).

### Iron metabolism markers

#### Hemoglobin

Hemoglobin (Hb) levels showed significant decrease in BCPs group either prior to or after RT when compared to healthy volunteers (p1 = 0.002 and < 0.000 respectively). Insignificant difference was found in Hb levels between pre- and post-RT (p2 = 0.149) (Table 2).

#### Ferroportin

Serum ferroportin showed significant increase before RT in BCPs when compared to healthy controls (p1 = 0.014). It significantly decreased after RT (p2 < 0.001) and became within normal control levels (p1 = 0.740) (Table 2).

#### Iron

Serum iron showed insignificant difference either prior to or following RT in BCPs group in comparison to healthy volunteers (p1 = 0.177 and = 0.305 respectively). However, iron levels significantly increased after RT when compared to before treatment (p2 < 0.001) (Table 2).

#### Ferritin

Ferritin levels showed significant increase prior to RT in BCPs group and healthy volunteers (p1 = 0.028) with insignificant difference after RT (p1 = 0.332). However, it significantly decreased after RT treatment when compared to before treatment (p2 < 0.001) (Table 2).

#### Transferrin saturation (%)

Transferrin saturation (%) showed insignificant difference either prior to or following RT in BCPs group in comparison to healthy volunteers (p1 = 0.197 and = 0.476 respectively). However, transferrin saturation (%) significantly increased after RT treatment when compared to before treatment (p2 < 0.001) (Table 2).

#### UIBC and TIBC

UIBC and TIBC showed insignificant difference either prior to or following RT in BCPs group in comparison to healthy volunteers. Moreover, their levels prior to and following RT revealed insignificant difference (Table 2).

## Discussion

The role of RT in the treatment of BC has long been recognized. Adjuvant RT reduces the risk of local recurrence following surgery and improves patients’ overall survival. The importance of RT in the treatment of BC may be the most clear in that it would not be possible to cure it without this type of adjuvant therapy. In many circumstances, breast sparing surgery would be impossible [13–15].

Ferroptosis is a newly discovered regulated cell death that depends on the peroxidation of cell membrane lipids in the presence of iron and differs in morphology and pathways from other forms of cellular death such as necroptosis, apoptosis, and autophagy. The hazardous accumulation of lipid peroxides in membranes of cells subsequently destroys membrane integrity, resulting in ferroptosis [16]. Recent research has discovered that IR causes ferroptotic cell death and that ferroptosis is a key component of RT-facilitated anticancer effects [1].

The present study discovered a substantial increase in serum levels of PTGS2 in patients after RT compared to the controls. As previously stated [17], this study found ferroptosis is linked to elevated PTGS2 expression. It should be highlighted that the effects of IR on lipid peroxidation and PTGS2 induction in the cell lines investigated by Lei et al. 2020 [2] were even more significant than those of most ferroptosis inducers, adding to the notion that IR is a strong ferroptosis inducer. Their data suggested that irradiation induces ferroptosis through increasing ROS production.

Our findings confirmed that there was a significant increase in MDA and GSH depletion after radiotherapy whether compared to pre radiotherapy or controls, indicating that radiotherapy induced lipid peroxidation which stimulates ferroptosis. Lei et al. 2020 [2] added that inhibition of ferroptosis promotes radio-resistance in malignant cells and that irradiation induces DNA damage, which further strengthens the fact that irradiation is a ferroptosis inducer. The increased ROS production arises from the excessive metabolic demands of the malignant cells to support the biomass accumulation and tumor growth compared to normal cells [18]. The high energy IR induces direct DNA damage [19], its indirect effects are accumulation of oxidases, and hydrolysis of cellular water which induces reduced glutathione depletion and ROS generation [20]. Fujihara et al. 2021 [21] reported that two processes cause ferroptosis: either by depleting the cellular antioxidant GSH or by direct inhibition of glutathione peroxidase 4 (GPX4) which reverses lipid oxidation. Song et al. 2020 [22] found that following GPX4 silencing, MDA levels increased considerably, GSH levels diminished, and ROS generation significantly elevated in the human TNBC cell lines MDA-MB-231 and HS578T. As a result, it was found that radiation causes GSH depletion, GPX4 dysfunction, and ROS generation, as well as ferroptotic cell death. In addition, we found a significant negative correlation between reduced glutathione level and increased MDA level. These finding directs us to improve the efficacy of radiotherapy or abrogate malignant cell resistance by regulating ferroptosis triggering. In accordance to our findings, Wu et al. (2020) [18] reported that the efficacy of RT increases when glutathione is depleted.

The role played by iron in cancer biology is complex where excess iron in cells is toxic and is linked to cancerous transformation, tumor progression, immune escape, and drug resistance [23]. Due to its ability to make lipid peroxides in an auto-amplifying way through the Fenton reaction, iron is involved in ferroptosis [24]. Our results showed, statistically increased serum iron after radiotherapy was observed in the patients versus its pre radiotherapy level. The membrane of red blood cells loses its integrity when it is exposed to IR which causes leakage of hemoglobin. Exposure to IR also leads to hemolysis through the lipid peroxidation process, which results in higher serum iron concentrations [25].

Recently, iron overload induces ferroptosis which is a regulated form of cell death. Ferroptosis leads to tumor suppression. Li et al. 2020 [26] stated that unraveling the regulatory mechanisms and genes involved in ferroptosis is a pre-requisite to develop strategies for targeting it in cancer therapy. In our study, the increased iron level post-RT is an inducer of ferroptosis as ferroptosis is prevented by sequestration of free iron or by scavenging ROS. This coincides with the findings of Wu et al. (2020) [18], while Christiansen et al. (2007) [19] contradicted our results as they observed decreased serum iron level post-RT. This might be due to that ionizing radiation was directly applied to the liver or isolated rat hepatocytes.

We found that, there was a significant increase in the percentage of saturation of serum transferrin which especially noticed after irradiation. This agrees with Brown et al. (2020) [23] who discovered that tumor cells trigger transferrin production to aid iron transport into the tumor microenvironment.

We detected that, serum levels of ferroportin were significantly higher in patients than the controls. After radiotherapy, its level decreased significantly in patients versus pre radiotherapy. This highlights the impact of radiation on ferroptosis. Silencing ferroportin increases the cellular labile iron pool and lipid peroxidation, thereby sensitizing cells towards ferroptosis [27]. Geng et al. 2018 [28] found knockdown of ferroportin accelerated erastin-induced ferroptosis by increasing iron-dependent lipid ROS accumulation, highlighting ferroportin as a potential therapeutic target site for neuroblastoma.

In the current study, serum ferritin level declined significantly after IR while iron levels significantly increased and iron overload induced by radiotherapy significantly stimulates ferroptosis [29]. Intracellular iron storage function is carried out by ferritin, which is structurally composed of 24 subunits of light (FTL) and heavy chains (FTH) that form a nano-cage complex to hold up to 4500 iron atoms. Ferritin sequesters excess intracellular iron and stores it in a redox-inactive form for future use in conditions of deficiency or high demand. Cellular and systemic ferritin levels are not only crucial indicators of iron status but are also important markers of inflammatory, immunological, and malignant disorders [30]. Decreasing of ferritin increases the cellular iron levels, leading to accumulation of ROS and ultimately cell death by ferroptosis [31]. This goes in line with Mou et al. (2019) [32] who forwarded an explanation for this phenomenon. They reported that the activation of autophagy can degrade ferritin and trigger ferroptosis in cancer cells. They added that ferritinophagy is crucial to induce ferroptosis as autophagy induces ferroptosis by generating lysosomal ROS and producing labile iron.

Finally, ferroptosis is a new cancer treatment target. Yet, the mechanisms that regulate it are complex and need to be deeply explored notably as regards its inducers or its inhibitors. Reducing cancer capacity to evade cell death by ferroptosis is a potential therapeutic strategy especially in tumor resistance to apoptosis.

Limitations of this study are small sample size and lack of information about use of hematinic by patients which may interfere with iron profile measurement and its interpretation. Also lack of data correlating our results with any post-treatment parameters like tumor control, progression-free, and overall survival. Future follow-up large-scale study is needed to confirm our finding.

## Conclusion

- Radiotherapy induced ferroptosis in breast cancer patients as a new cell death mechanism and PTGS2 is a biomarker of ferroptosis.
- Iron modulation is a useful approach for the treatment of BC especially if combined with targeted therapy and immune based therapy.
- Further studies are warranted to be translated into clinical compounds.

## Data Availability

The datasets used and/or analyzed during the current study are available from the corresponding author on reasonable request.

## Abbreviations

RT: Radiotherapy
GSH: Reduced glutathione
MDA: Malondialdehyde
PTGS2: Prostaglandin-endoperoxide synthase 2
ROS: Reactive oxygen species
UIBC: Unsaturated iron binding capacity
TIBC: Total iron binding capacity
ER: Estrogen receptor
PR: Progesterone receptor
